# Structural performance assessment of high-strength concrete beams using terrestrial laser scanning and finite element analysis

**DOI:** 10.1038/s41598-026-68044-1

**Published:** 2026-09-03

**Authors:** Ahmed E. Sedawy, Ashraf G. Shehata, Ashraf A. A. Beshr

**Affiliations:** 1 Department of Civil Engineering, Misr Higher Institute for Engineering and Technology, Mansoura, Egypt; 2 Civil Engineering Department, Delta University for Science and Technology, Gamasa, Egypt; 3 Surveying and Photogrammetry, Public Works Department, Faculty of Engineering, Mansoura University, Mansoura, 35516 Egypt

**Keywords:** High-strength concrete (HSC), Terrestrial laser scanning (TLS), Deflection monitoring, Finite element method (FEM), Engineering, Materials science

## Abstract

High-strength concrete (HSC) is widely used in modern structures because of its superior mechanical performance; however, accurate deflection monitoring is essential for assessing serviceability and validating numerical models. This study evaluates the capability of Terrestrial Laser Scanning (TLS) as a non-contact technique for measuring the deflection of reinforced concrete beams under static loading. The proposed methodology is primarily intended for laboratory-scale validation but provides a transferable framework for future field applications and structural health monitoring. Experimental TLS measurements were compared with dial gauge and total station measurements, analytical calculations, and finite element simulations using ABAQUS and ANSYS. The results showed excellent agreement between TLS and conventional techniques. At the ultimate load of 230 kN, the measured mid-span deflections were 26.0 mm using TLS, 26.3 mm using the dial gauge, and 26.1 mm using ABAQUS, while ANSYS predicted 21.8 mm. Besides providing accurate deflection measurements, TLS enabled rapid, full-field deformation mapping without physical contact. The close agreement between experimental and numerical results confirms the reliability of TLS for structural deformation monitoring and numerical model validation. Integrating TLS with finite element analysis offers an effective framework for structural performance assessment and supports future structural health monitoring applications. The present investigation was limited to static four-point bending tests. Nevertheless, the proposed TLS-based monitoring methodology is independent of the loading configuration and can potentially be extended to beams subjected to concentrated loads, moving loads, cyclic loading, or other structural loading scenarios, provided that appropriate data acquisition procedures are adopted. Experimental verification under these loading conditions is recommended for future research.

## Introduction

The accurate assessment and prediction of deflection in high-strength concrete (HSC) elements are fundamental requirements for ensuring the serviceability, safety, and long-term performance of modern reinforced concrete structures. Owing to its high compressive strength (typically exceeding 60 MPa), superior durability, and enhanced load-carrying capacity, HSC has been extensively employed in high-rise buildings, long-span bridges, and other heavily loaded structural systems. Nevertheless, compared with conventional concrete, HSC generally exhibits lower ductility and a more brittle failure mechanism, making deformation monitoring particularly important under both service and ultimate loading conditions. Excessive deflection may adversely affect structural functionality, induce cracking, reduce user comfort, and compromise long-term structural performance. Consequently, accurate deflection measurement is essential for evaluating structural behavior, validating analytical and numerical models, and ensuring compliance with design code requirements.

Terrestrial Laser Scanning (TLS) has become a well-established non-contact measurement technology in surveying, civil engineering, and structural monitoring over the past two decades. The technology enables rapid acquisition of dense three-dimensional point clouds, allowing comprehensive geometric documentation and full-field deformation assessment without direct physical contact with the structure. Unlike conventional displacement measurement techniques, TLS provides continuous spatial information over the entire structural surface, making it particularly attractive for deformation monitoring and structural health assessment. Although TLS itself is not a new technology, its application for quantitative validation of reinforced concrete beam deflections through integration with conventional experimental measurements, analytical calculations, and finite element simulations remains relatively limited. Therefore, the novelty of the present study lies not in the use of TLS itself, but in the development of an integrated structural assessment framework combining experimental observations, numerical simulations, and conventional monitoring techniques for comprehensive validation of beam behavior.

Structural Health Monitoring (SHM) is essential for maintaining both the safety and serviceability of civil engineering structures throughout their operational life^1^. Conventional inspection and monitoring techniques generally rely on discrete sensors or physical targets installed at selected locations on the structure. Consequently, deformation measurements are restricted to a limited number of monitoring points, requiring prior knowledge of expected deformation zones. In addition, the installation of contact sensors may interfere with the testing procedure or become susceptible to damage during loading^2^. Previous studies have successfully compared deformation measurements obtained using photogrammetric techniques, one-dimensional laser transducers, and terrestrial laser scanners, demonstrating the potential of non-contact optical measurement systems for structural monitoring^3^. Building upon these developments, the present study evaluates the three-dimensional deformation of reinforced concrete (RC) beams subjected to different loading stages and compares TLS measurements with conventional surveying instruments, analytical calculations, finite element simulations, and dial gauge observations.

TLS effectively addresses many limitations associated with conventional monitoring systems by enabling rapid, non-contact, high-density deformation measurements for both laboratory-scale specimens and full-scale engineering structures. During the past decade, laser scanning and advanced photogrammetric techniques have been increasingly integrated into structural health monitoring applications because of their capability to provide complete three-dimensional surface information while eliminating the need for physical contact with the monitored structure. Furthermore, the redundancy of dense point-cloud data enables reliable deformation estimation and geometric reconstruction using least-squares adjustment techniques, thereby improving measurement reliability and facilitating quantitative assessment of structural performance^4^.

Alongside experimental measurements, numerical simulation has become an indispensable tool for investigating the nonlinear response of reinforced concrete structures. Finite Element Analysis (FEA), implemented using advanced software packages such as ANSYS and ABAQUS, enables accurate simulation of concrete cracking, crushing, nonlinear material behavior, and reinforcement interaction under external loading^5,6^. In the present study, numerical models were developed to complement the experimental program by simulating the deflection response of reinforced concrete beams under identical loading conditions. ANSYS was employed using the SOLID65 element for nonlinear concrete analysis, whereas ABAQUS utilized the Concrete Damage Plasticity (CDP) model to simulate damage evolution^7–9^. Integrating TLS measurements with validated finite element models provides a comprehensive framework for structural performance assessment while enhancing confidence in both experimental observations and numerical predictions^10–12^.

The accuracy and reliability of the proposed TLS-based methodology were evaluated through quantitative comparison with conventional measurement techniques, including dial gauge and total station observations, together with analytical calculations and finite element simulations performed using ABAQUS and ANSYS. Validation was based on comparing beam deflections at identical loading levels, particularly at the ultimate loading stage. The agreement between TLS measurements and the independent reference methods was adopted as the primary performance indicator for evaluating the reliability of the proposed structural monitoring methodology.

Although advanced cementitious materials such as Ultra-High-Performance Concrete (UHPC) have recently received considerable research attention, conventional HSC continues to be one of the most widely adopted construction materials because of its favorable balance between strength, durability, availability, and construction cost. Accordingly, HSC was selected in this investigation as a representative structural material for validating the proposed TLS-based monitoring methodology. Nevertheless, the developed framework is material-independent and can readily be extended to UHPC and other advanced cement-based materials in future investigations.

Unlike previous investigations that primarily focused on geometric documentation or isolated deformation measurements, the present research proposes an integrated structural assessment framework that combines TLS measurements, conventional experimental techniques, analytical calculations, and nonlinear finite element simulations to evaluate the structural response of reinforced concrete beams subjected to static loading. This comprehensive validation methodology provides reliable experimental and numerical verification of beam deflection and demonstrates the applicability of TLS as an effective tool for structural deformation monitoring and finite element model validation.

## Literature review and research gap

Structural Health Monitoring (SHM) has become an essential component of modern infrastructure management, providing reliable information for evaluating structural safety, durability, and serviceability throughout the service life of reinforced concrete (RC) structures^13–15^. Conventional monitoring techniques, including dial gauges, strain gauges, and displacement transducers, have long been adopted for deformation measurements. However, these methods are generally limited to discrete measurement locations, require physical contact with the structure, and may be affected by installation complexity and environmental conditions, reducing their effectiveness for comprehensive structural assessment^16^.

Recent advances in Terrestrial Laser Scanning (TLS) have significantly expanded the capabilities of non-contact structural monitoring. TLS enables rapid acquisition of dense three-dimensional point clouds, allowing full-field deformation measurements with high spatial resolution while eliminating the need for extensive physical instrumentation. Consequently, TLS has been successfully applied in numerous civil engineering applications, including three-dimensional modeling, bridge monitoring, industrial facility inspection, and documentation of historical structures^17–21^. Furthermore, the redundancy of point-cloud data facilitates robust deformation analysis and accuracy assessment through advanced registration and least-squares adjustment techniques^22^.

Parallel developments in computational mechanics have established Finite Element Analysis (FEA) as an indispensable tool for predicting the nonlinear behavior of reinforced concrete structures. Advanced constitutive models implemented in ABAQUS and ANSYS, particularly the Concrete Damage Plasticity (CDP) model and nonlinear concrete material formulations, have demonstrated excellent capability for simulating cracking, crushing, stiffness degradation, and structural deformation under external loading^23–25^. Previous investigations have consistently reported good agreement between numerical simulations and experimental observations, confirming the reliability of these computational approaches for structural performance assessment.

Despite these advances, relatively few studies have developed an integrated framework that quantitatively combines TLS measurements, conventional experimental techniques, analytical calculations, and nonlinear finite element simulations for validating the structural behavior of high-strength reinforced concrete beams. Most existing investigations have focused either on deformation monitoring or numerical simulation separately, while comprehensive validation involving multiple independent measurement techniques remains limited, particularly for HSC beams subjected to incremental static loading^26^.

Accordingly, the present study addresses this research gap by developing and validating an integrated structural assessment methodology that combines TLS measurements with conventional surveying techniques, analytical calculations, dial gauge observations, and finite element simulations performed using ABAQUS and ANSYS. The proposed framework provides a comprehensive evaluation of beam deflection while establishing the reliability of TLS as a practical tool for structural deformation monitoring and numerical model verification.

## Experimental program for R.C beams

**Fig. 1:**
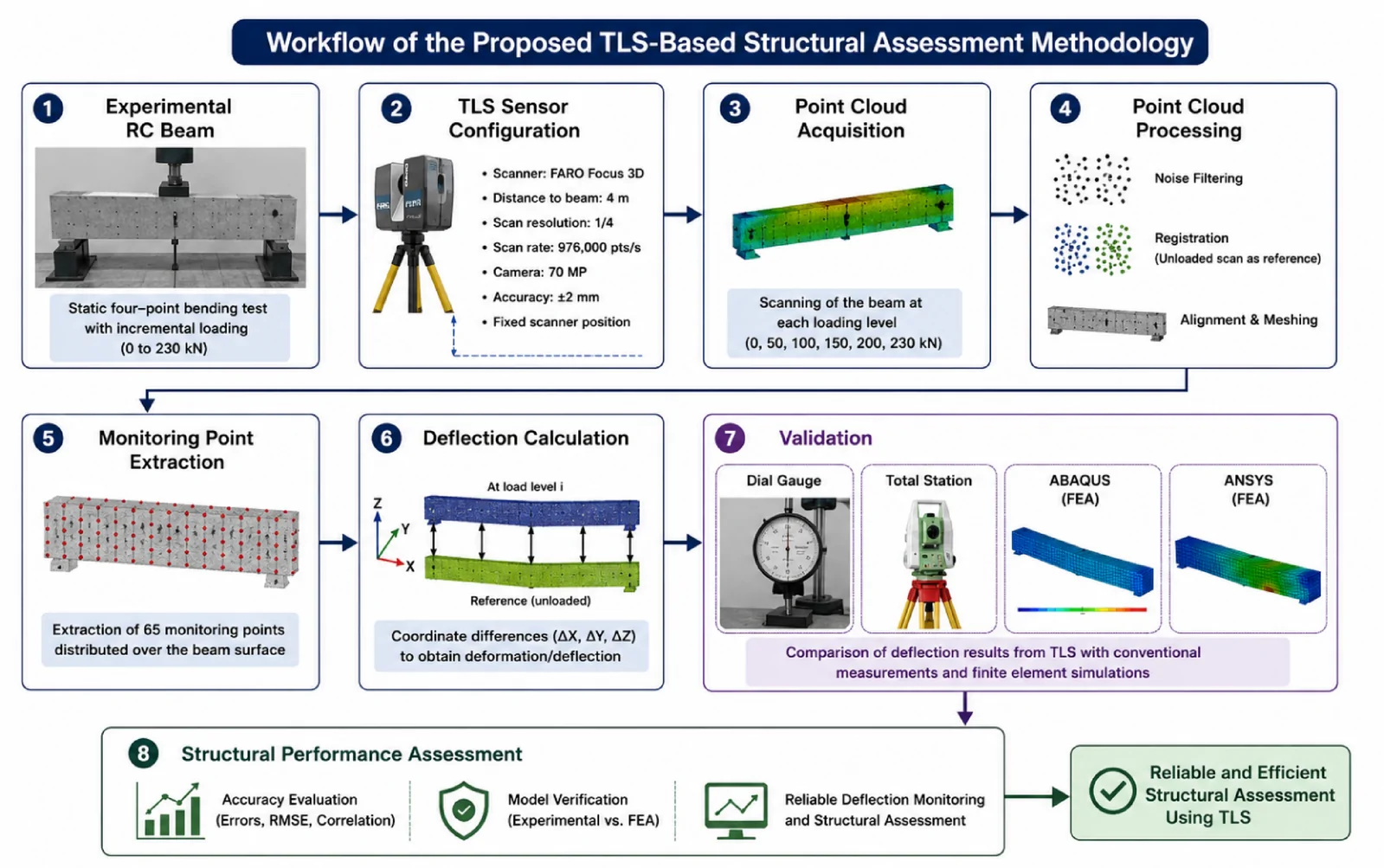
Workflow of the proposed TLS-based structural assessment methodology

To improve the clarity of the proposed methodology, Fig. 1 presents the complete workflow adopted in this study. The workflow summarizes the successive stages starting from TLS data acquisition, point cloud preprocessing, registration and filtering, extraction of monitoring points, deformation calculation, and finally validation against conventional measurements and finite element simulations. This workflow provides a comprehensive overview of the integration of TLS within the proposed structural assessment framework.

**Fig. 2:**
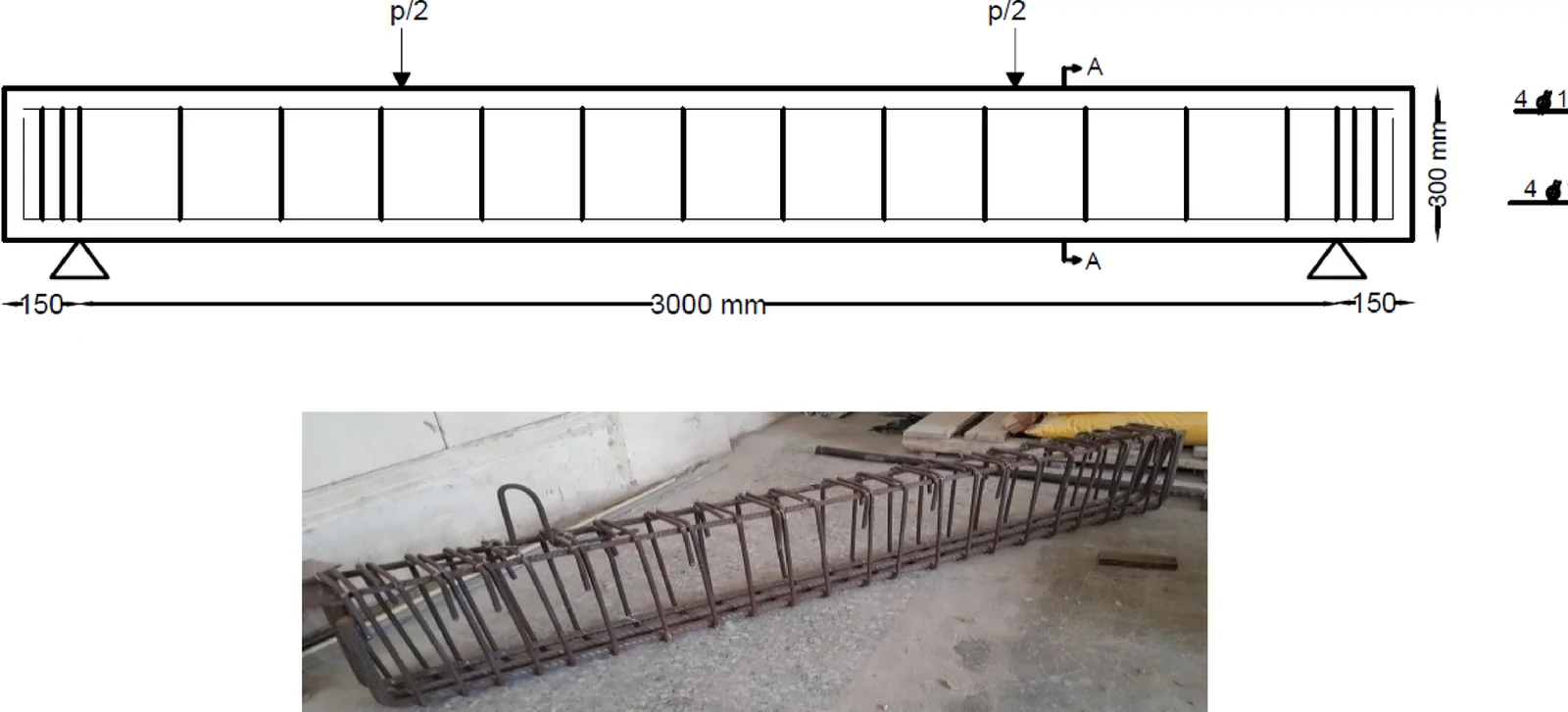
Details of beam reinforcement

In this section, concrete beams, their sections, and reinforcement are explained in detail, as this is the part on which the practical test will be conducted. Four concrete beams were poured with the same section and reinforcement. The reinforced concrete beam was designed in accordance with ACI 318-19. Each beam length is 3 m with a cross-section of (30 cm × 30 cm) with 4Φ12 upper and 4Φ16 lower reinforcement bars made of high tensile steel. The details of the tested specimens are shown in Fig. 2. Stirrups are distributed 5Φ8/m'. A high-strength concrete mix is employed. Concrete mix used to cast the tested RC beams with concrete compressive strength equal to 61 MPa consisted of Portland cement, natural aggregates, natural water andPowder silica smolder with 92% SiO2, particular gravity of 2.2^13,14^. Super plasticizers are used to improve the solid characteristics. Mixing was performed using a concrete tilted rotating drum mixer with maximum capacity 0.125 m3. Sand, dolomite and cement were dry mixed. The water was then gradually added while mixing continued for an additional two minutes, resulting in a homogeneous concrete mixture. The concrete was cast in the molds and cured by covering the specimens with moist burlap sheets until testing.

**Fig. 3:**
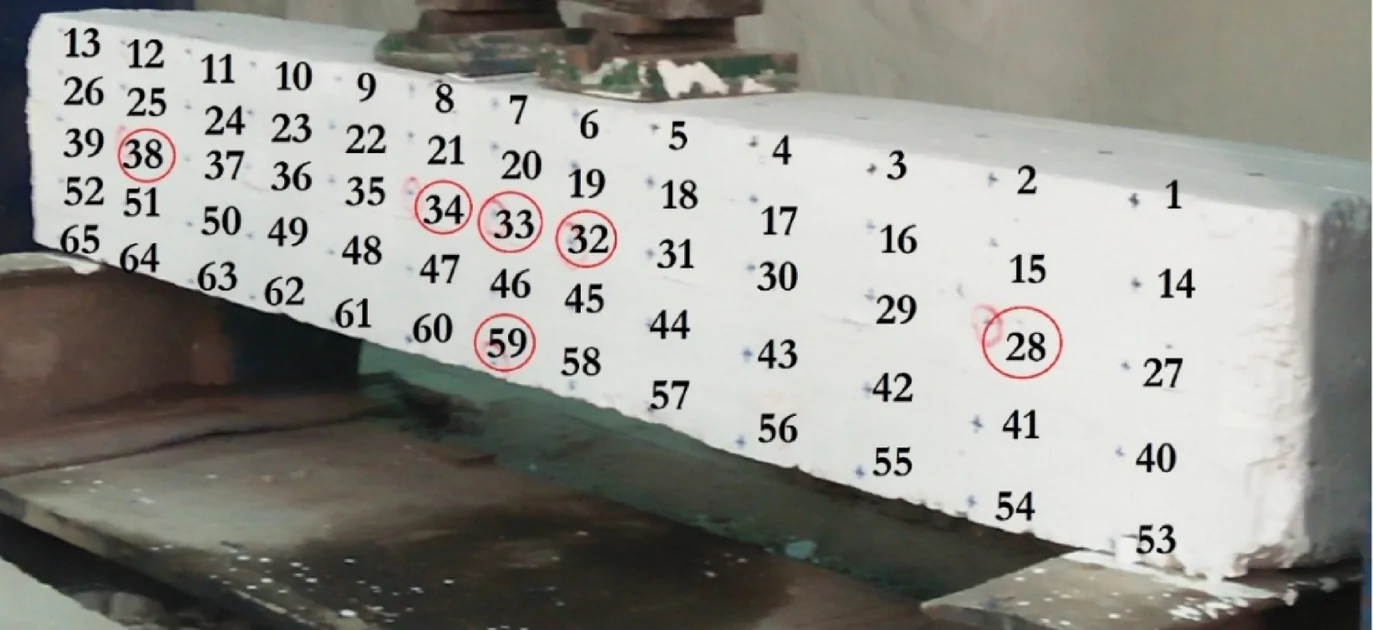
Monitoring points on the R.C beam face

TLS is used to cllect geodetic date (3D Fig. of studied beam and coordinates of monitoring points on beam façade) where the beam face is divided into 65 monitoring points distributed to cover the whole surface of beam, as appeared in Fig. 3. By knowing the coordinates of these points (65 monitoring points on the outer surface of the beam) with each load that is applied, the actual deformation shape of the concrete beam is accurately drawn. The primary objective of this experiment is to monitor deformations resulting from the applied loads. The scope of the study includes acquiring reliable data before and after each load application, which was compared with three different theoretical and geotechnical techniques (ABAQUS, ANSYS, and dial gauge). After the raw point cloud data are imported into the processing software, preprocessing steps are performed, including noise removal, filtering, edge detection, and the addition of supplementary information (monitoring point coordinates for each load) to the raw grid for subsequent processing stages such as registration and meshing. Magnet Collage software was used for TLS data processing and creating a 3D model of the studied beams.

Utilizing high-density scanning and the advanced tools available in the processing software, registration is performed between the initial scan at zero load, used as a reference, and subsequent scans after each loading stage. The average registration error was approximately 0.0001 m, which ensured that the beam’s position remained consistent across all load cases within this tolerance range. The points of interest for deformation monitoring can be digitized and exported as a list for further analysis. These 3D points can be exported for the initial case before the start of the loading test and after each loading case, allowing for comparisons of the results after applying each load with the initial results and with previous loading cases.

A four-point bending configuration was selected because it provides a constant bending moment region between the two loading points while minimizing shear effects within the central span. This loading arrangement enables accurate measurement of beam deflection and facilitates reliable comparison between experimental observations and finite element predictions. It is also one of the most commonly adopted configurations for evaluating the flexural behavior of reinforced concrete beams.

## Experimental work using TLS

**Fig. 4:**
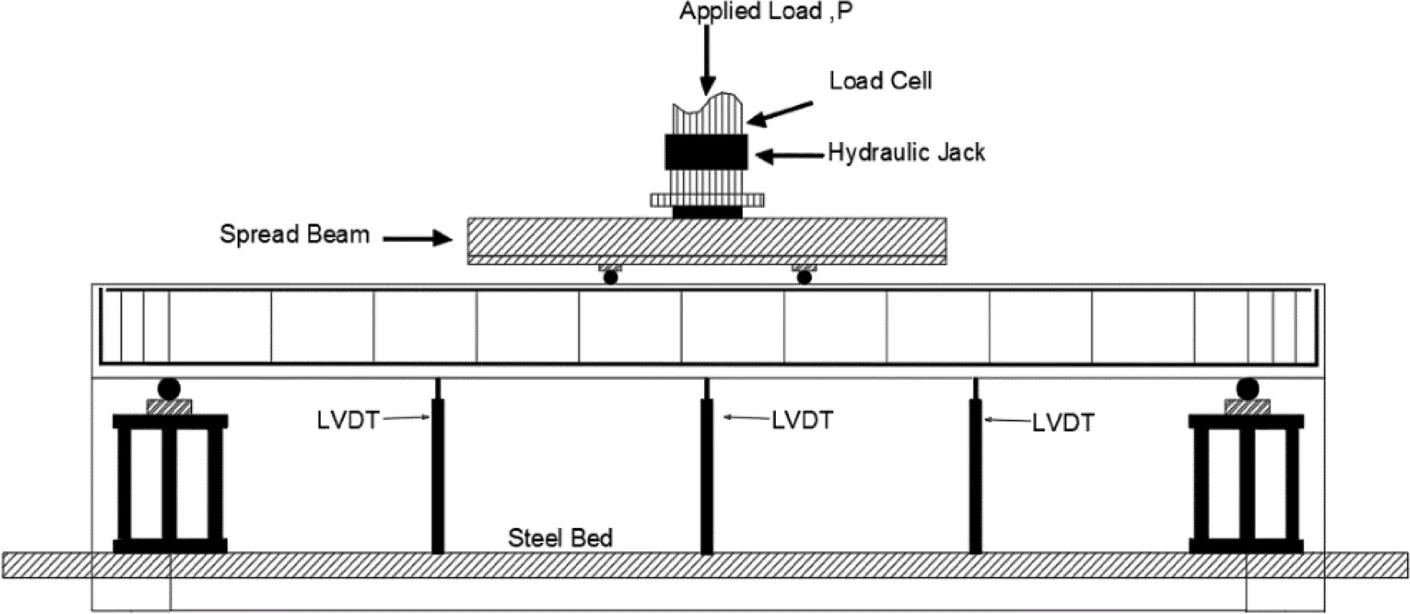
Sketch of experimental test setup

The beams were tested using a manually operated hydraulic jack with a capacity of 1000 kN, powered by an oil pump. Figure 4 illustrates the test setup and the instrumentation used in the experiment. A digital load indicator connected to an electrical load cell with a precision of 0.1 kN was used to monitor the applied vertical loads. Deflections of the tested beams were recorded using three electric dial gauges (LVDT): one positioned at the mid-span, and the other two placed at the quarter-span points from each support on the bottom face of the beam.

**Fig. 5:**
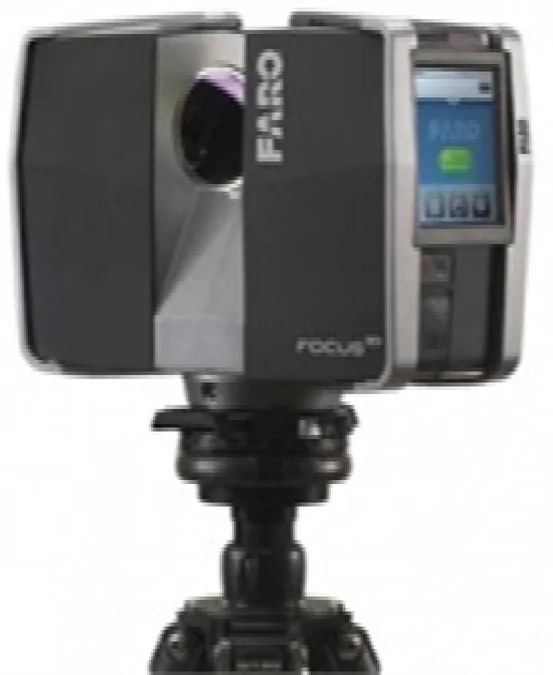
Faro3Dlaserscanner

The TLS system used in this study is shown in Fig. 5.The scanner operates within a range of 0.6 m up to 120 m, with an accuracy of ± 2 mm for distances between 10 and 25 m. It provides a reflectance rate of 90% for objects sized between 0.6 and 10 mm, and 10% for objects between 2.2 and 25 mm. The angular resolution for both vertical and horizontal directions is set at 0.009°. The system scans at a rate of approximately 976,000 points per second and is lightweight for ease of use. It is equipped with an advanced shading camera boasting a resolution of 70 megapixels^18,27^. Some laboratory tests were carried out before field observations and data collection using an applied laser scanner to ensure the accuracy of the device mentioned in the specification. The optimum positions for the instruments were chosen in order to save time and effort (4 m from tested beams). For each loading case of the studied beam, a scan by TLS is taken for the concrete beam from the same position occupied by TLS. The position of TLS was not changed at all load cases during the experiment, and all scans are taken for all loading cases with the same TLS resolution.

The scanning process generates a dense 3D point cloud with coordinates in the x, y, and z directions. After data acquisition, the raw point clouds undergo a series of filtering and cleaning procedures designed to extract and enhance the relevant data required for subsequent processing.

Although the manufacturer specifies an absolute ranging accuracy of approximately ± 2 mm for the FARO Focus 3D scanner, this value refers to single-point absolute positioning under general operating conditions. In deformation monitoring applications, displacement is determined from the relative coordinate differences between repeated scans acquired using identical scanner geometry and registration procedures. Consequently, the relative accuracy of displacement measurements is governed primarily by registration quality and point cloud processing rather than the absolute ranging accuracy of individual points. TLS is used to quantify and model the three-dimensional deformations of the beams under applied loading.

**Fig. 6:**
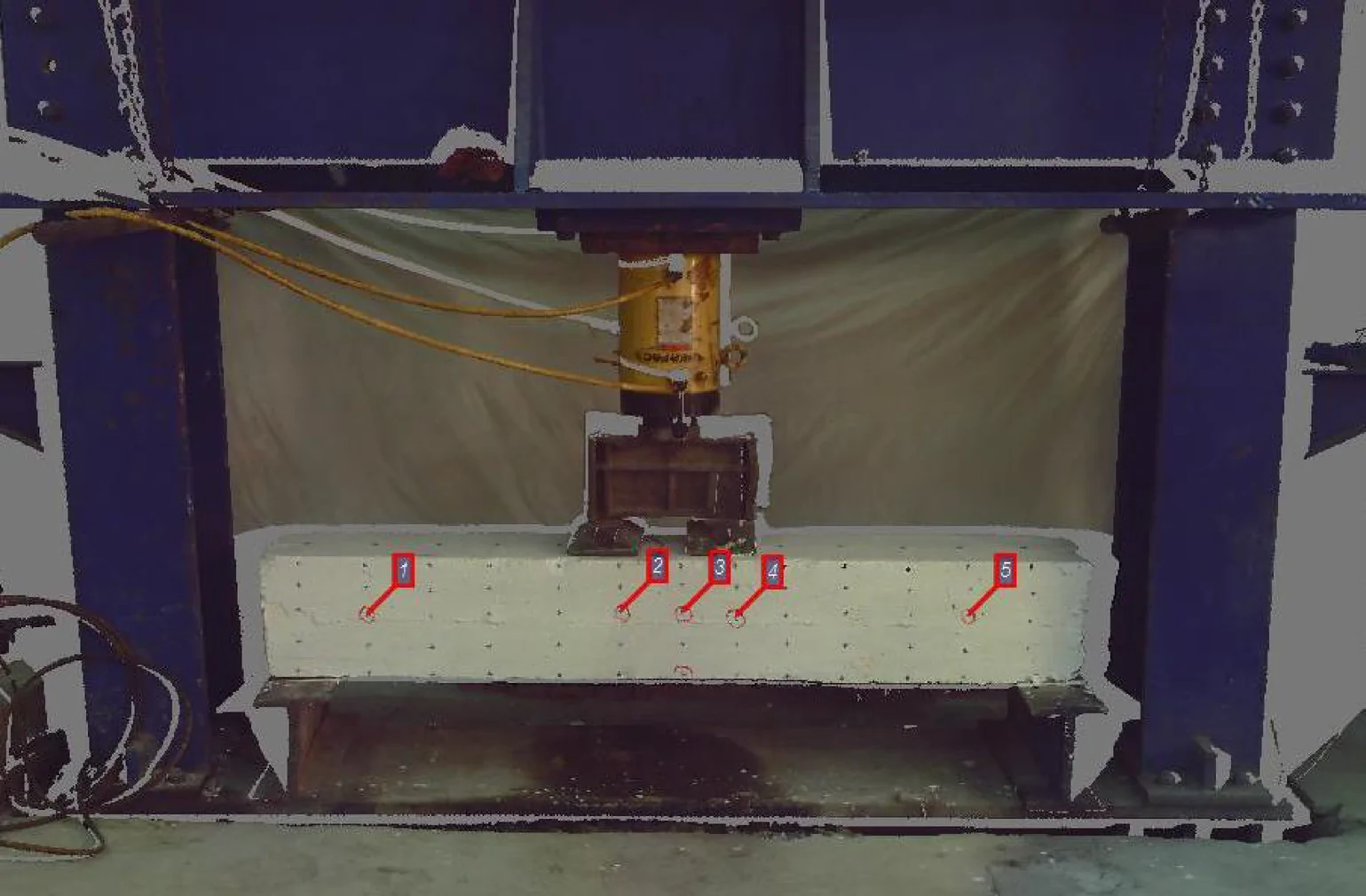
Points to be monitored with the RC Beam

**Fig. 7:**
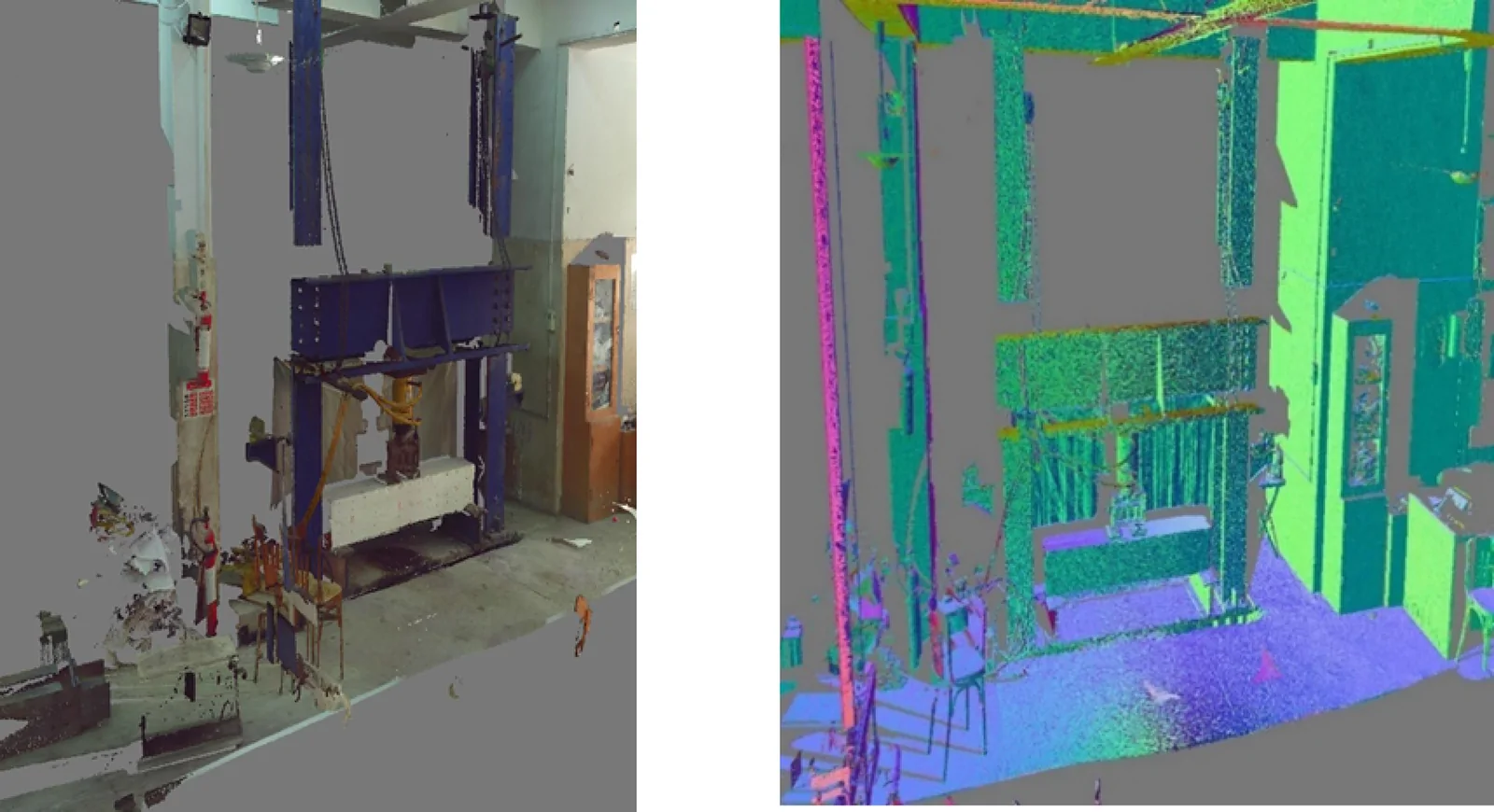
RC beam from point cloud of TLS

Using the TLS process, the longitudinal distribution of the collected points provided a wide coverage of the beam^19,28^, as illustrated in Figs. 6 and 7. The selected observation points correspond to locations where the largest deformation values were expected and identified. These monitoring points were also monitored by using a Total Station to compare the results. The TLS-based monitoring procedure consisted of six sequential stages. First, the scanner was positioned at a fixed distance of approximately 4 m from the beam to ensure identical acquisition geometry throughout all loading stages. Second, point clouds were acquired for the unloaded beam and after each loading increment. Third, the acquired point clouds were processed through filtering and noise removal, followed by point cloud registration using the unloaded scan as the reference dataset. Fourth, the registered point clouds were used to extract the coordinates of the 65 monitoring points distributed over the beam surface.

The rationale for selecting TLS in this study was its ability to provide non-contact, three-dimensional, and spatially distributed measurements of beam deformation. Unlike the dial gauge, which provides displacement at a localized measurement point, and the total station, which relies on predefined observation points, TLS enables measurements to be obtained from multiple points distributed over the beam surface. This capability allows the spatial deformation pattern to be evaluated in addition to the maximum mid-span deflection. Therefore, TLS was used in this study as an independent full-field measurement technique and not as a replacement for conventional measurement methods. Its performance was evaluated through direct comparison with dial gauge and total station measurements, theoretical calculations, and finite element predictions.

The smallest measured displacement increments should not be interpreted solely based on the nominal absolute accuracy of the scanner. Instead, they result from relative comparisons between repeated scans acquired under identical experimental conditions. The agreement observed with dial gauge measurements further confirms that the measured deflections represent actual structural behavior rather than measurement noise. Fifth, coordinate differences between successive loading stages were calculated to obtain the beam deformation field. Finally, the measured deformations were validated by comparison with dial gauge measurements, total station observations, and finite element predictions obtained from ABAQUS and ANSYS.

### Measurement uncertainty and data quality assessment

Measurement uncertainty was considered from both the experimental and numerical perspectives. For the TLS measurements, the main potential sources of uncertainty were related to scanner positioning and registration, surface reflectivity, scanning geometry, environmental conditions, and point-cloud processing. For the conventional measurements, uncertainty may arise from instrument resolution, target positioning, and manual reading of the dial gauge. The theoretical and finite element predictions are additionally affected by assumptions related to material properties, constitutive models, boundary conditions, and representation of cracking and nonlinear behavior.

To reduce the influence of these sources of uncertainty, the same reference coordinate system, loading stages, and measurement locations were maintained throughout the experimental program. The TLS point clouds were processed using the same registration and deformation-extraction procedure for all loading stages. The comparison with the dial gauge and total station measurements provided independent experimental references, while the theoretical and finite element results provided independent analytical and numerical comparisons. Accordingly, the reliability of the TLS measurements was not assessed using a single measurement technique, but through comparison with multiple independent sources.

The unloaded scan (P = 0 kN) was adopted as the reference configuration for all deformation measurements. Consequently, beam deflections were determined from the relative coordinate differences between the reference scan and the scans acquired at each loading stage rather than from independent absolute coordinate measurements. This relative measurement approach significantly reduces the influence of systematic positioning errors and enhances the reliability of deformation monitoring.

The reliability of the proposed TLS-based methodology was further verified through comparison with conventional measurement techniques, including dial gauge and total station observations, as well as finite element simulations developed using ABAQUS and ANSYS. The close agreement observed among these independent methods indicates that the measured deflections primarily represent the actual structural response of the beam rather than random fluctuations associated with point cloud noise.

It should be noted that the present investigation was conducted under controlled laboratory conditions and focused on static four-point bending of reinforced concrete beams. Therefore, the uncertainty assessment should not be interpreted as a complete probabilistic uncertainty quantification for all possible field applications. Instead, the analysis evaluates the principal sources of measurement and modeling uncertainty relevant to the present experimental configuration. Factors such as environmental variability, scanner-to-target distance, surface characteristics, registration quality, and modeling assumptions may influence the measured or predicted deformation. These factors represent potential sources of uncertainty that should be considered when extending the proposed TLS-based methodology to larger structural systems or field applications.

## Test results and analysis

The adjusted coordinates of all monitoring points and its associated accuracy are calculated for all loadings cases from TLS observations.

**Fig. 8:**
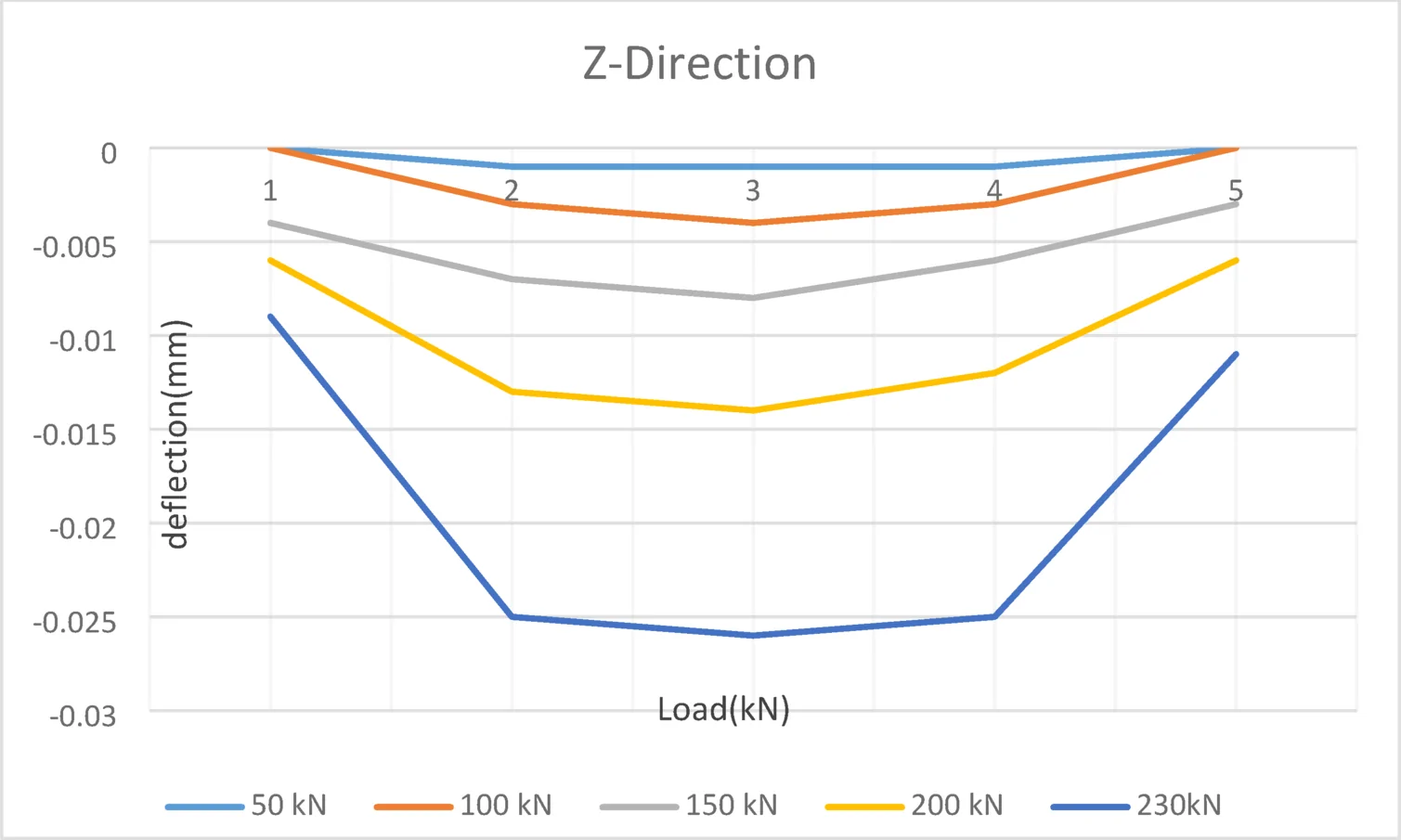
Beam monitoring for all case loading

Table 1 shows a sample output of the adjusted coordinates (for Load P = 0.0kN)and Table 2 shows the values of the calculated coordinate differences for all loads. Point displacements in 3-D are derived by subtracting the adjusted coordinates from each loading instance and the coordinates acquired from the unloading case. From Fig. 8, it is clear that deflection is symmetrical, with the minimum displacement occurring at points 1 and 5 near the supports. The deflection increases progressively toward the center, reaching a maximum vertical displacement of 26 mm at mid-span (point 3) under the final load of 230 kN. To provide an independent reference for evaluating the measured deflection response, the observed beam deflections were assessed in relation to the serviceability requirements of ACI 318-19. The ACI provisions were considered as a design-oriented reference for deflection control and serviceability assessment. This comparison provides a recognized structural design basis for interpreting the magnitude of the measured deflections in addition to the experimental, theoretical, and finite element comparisons presented in this study. The code-based assessment is intended specifically to evaluate deflection control and does not constitute a complete verification of all strength and serviceability requirements for the tested beams.

**Table 1:** Initial adjusted coordinates of the monitoring points defining the reference configuration before loading (P = 0 kN).

Point ID	Coordinates					
	X. (mm)	σ_X_.(mm)	Y.(mm)	σy.(mm)	Z.(mm)	σz.(mm)
1	− 1069	0.01	2145	0.14	93,664	0.09
2	− 455	0.12	2239	0.21	93,677	0.02
3	− 305	0.23	2264	0.16	93,673	0.06
4	− 175	0.02	2285	0.18	93,661	0.03
5	386	0.17	2378	0.09	93,664	0.01

**Table 2:** Values of the calculated coordinate differences for all loads.

ID	Co-ordinates, m			Deformation , mm		
	X	Y	Z	∆X	∆Y	∆Z
(a) Coordinate differences for load (50 kN)						
1	− 1.0670	2.1460	93.6620	2.0	1.0	0.0
2	− 0.4540	2.2410	93.6760	1.0	2.0	− 1.0
3	− 0.3030	2.2650	93.6720	2.0	1.0	− 1.0
4	− 0.1760	2.2880	93.660	− 1.0	3.0	− 1.0
5	0.3870	2.3780	93.6650	1.0	0.0	0.0
(b) Coordinate differences for load (100 kN)						
1	− 1.0680	2.1450	93.6640	1.0	0.0	0.0
2	− 0.4550	2.2410	93.6740	0.0	2.0	− 3.0
3	− 0.3040	2.2630	93.6710	1.0	− 1.0	− 4.0
4	− 0.1770	2.2860	93.6580	− 2.0	1.0	− 3.0
5	0.3860	2.3790	93.6640	0.0	1.0	0.0
(c) Coordinate differences for load (150 kN)						
1	− 1.0670	2.1430	93.6600	2.0	− 2.0	− 4.0
2	− 0.4540	2.2390	93.6700	1.0	0.0	− 7.0
3	− 0.3040	2.2640	93.6660	1.0	0.0	− 8.0
4	− 0.1750	2.2860	93.6550	0.0	1.0	− 6.0
5	0.3860	2.3790	93.6610	0.0	1.0	− 3.0
(d) Coordinate differences for load (200 kN)						
1	− 1.0670	2.1420	93.6580	2.0	− 3.0	− 6.0
2	− 0.4530	2.2400	93.6630	2.0	1.0	− 13.0
3	− 0.3040	2.2650	93.6610	1.0	1.0	− 14.0
4	− 0.1760	2.2850	93.6480	− 1.0	0.0	− 12.0
5	0.3880	2.3810	93.6580	2.0	3.0	− 6.0
(e) Coordinate differences for load (230 kN)						
1	− 1.0690	2.1410	93.6550	0.0	− 4.0	− 9.0
2	− 0.4550	2.2400	93.6520	0.0	1.0	− 25.0
3	− 0.3030	2.2640	93.6470	2.0	0.0	− 26.0
4	− 0.1730	2.2870	93.6350	2.0	2.0	− 25.0
5	0.3880	2.3790	93.6530	2.0	1.0	− 11.0

This behavior reflects the typical flexural response of simply supported reinforced concrete beams, where the maximum bending moment and deflection occur at the span center.

The unloaded scan (P = 0 kN) was adopted as the reference configuration for all deformation analyses. The coordinates listed in Table 1 correspond to the reference geometry of the beam and are not displacement values. Structural deflections were subsequently computed as coordinate differences between each loaded scan and the reference scan.

## Finite element modeling

Two independent finite element platforms were employed to improve the robustness of the numerical investigation. ABAQUS was selected because of its advanced nonlinear constitutive modeling capabilities for reinforced concrete, particularly the Concrete Damaged Plasticity (CDP) model. ANSYS was additionally utilized as an independent verification platform to evaluate the consistency of the predicted structural response using a different finite element implementation and solution strategy. The comparison between both software packages increases confidence in the numerical results and reduces dependence on a single computational platform.

### Numerical analysis using ABAQUS

**Fig. 9:**
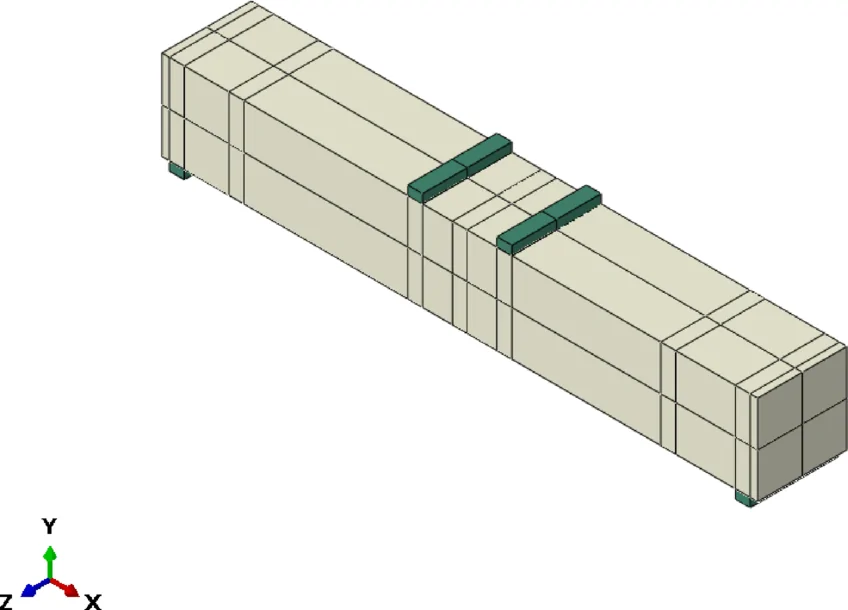
3D model for concrete beam

The finite element analysis was conducted using ABAQUS (2022), with both geometric and material nonlinearities addressed through the static general solver, as depicted in Fig. 9. In the finite element model, a global Cartesian coordinate system was adopted, where the X-axis represents the longitudinal direction of the beam, the Y-axis represents the lateral direction, and the Z-axis denotes the global vertical direction. According to ABAQUS notation, the translational degrees of freedom are defined as U1 (X-axis), U2 (Y-axis), and U3 (Z-axis). In this study, displacement control was applied along the Z-axis (U3), corresponding to the vertical loading direction.

### Element type and meshing

**Fig. 10:**
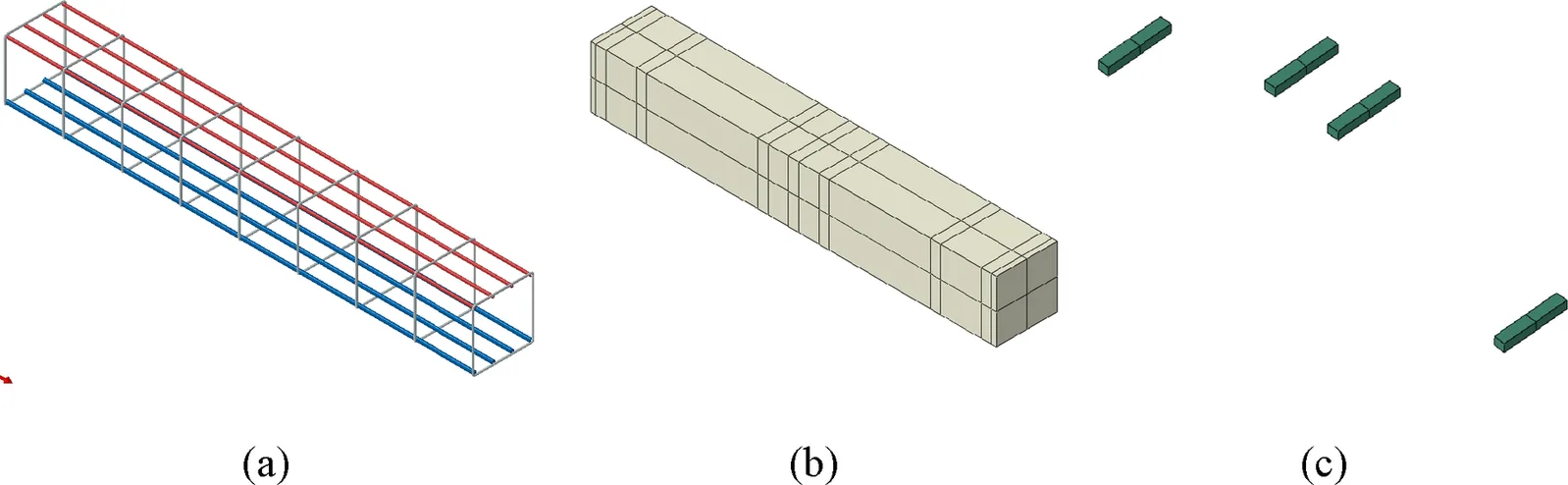
Basic components used in FE model: (**a**) reinforcement, (**b**) concrete, (**c**) pushing plates and supports

"The concrete beam was represented using solid elements of type C3D8R, while the reinforcement bars and stirrups were modelled using two-node linear truss elements (T3D2). A uniform mesh size of 40 mm was applied to all components, and Fig. 10 illustrates the meshing pattern used in the model.

### Contact modeling

Surface-to-surface contact formulations were applied to all interface regions. Normal interactions were enforced as hard contacts along the interaction planes, while tangential responses were governed by the penalty friction method. The friction coefficient between steel plates, supports, and the concrete beam was defined as 0.25, following recommendations from previous experimental and numerical studies^29^.This value is widely adopted in FE simulations to represent realistic steel–concrete interaction and to ensure numerical stability.”. Additionally, an embedded constraint technique was used to integrate steel reinforcement within the solid concrete components.

### Boundary conditions (BC) and loading

**Fig. 11:**
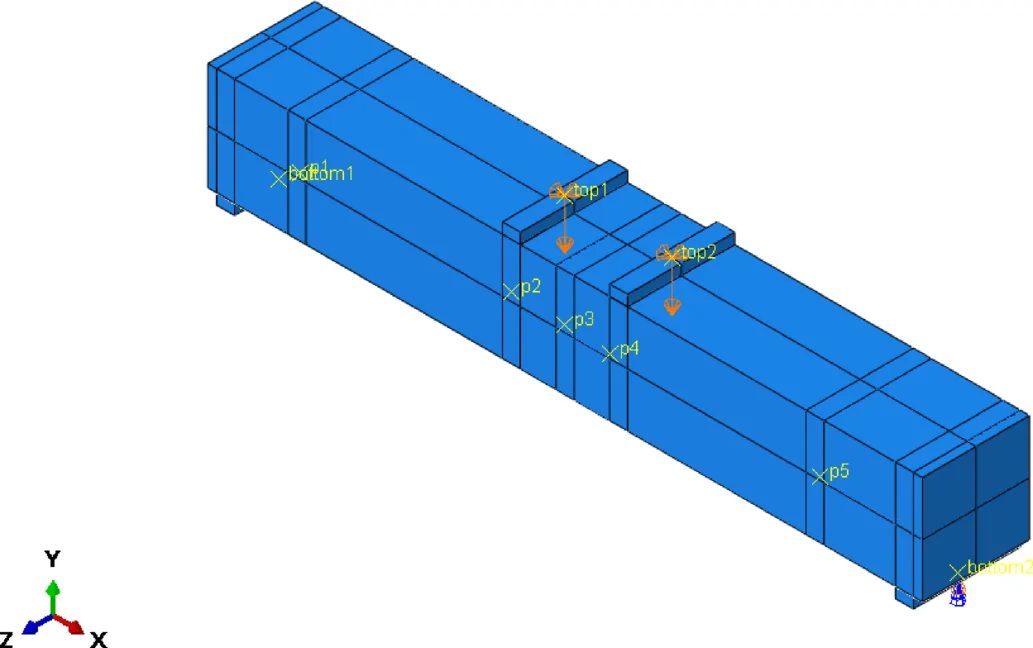
Boundary condition

"As shown in Fig. 11, the study utilized a detailed numerical model replicating the actual concrete beam specimens. Multi-point constraints were imposed on the entire bottom surface of the support plate, which acts as the bearing surface for the specimen, to define the boundary conditions. To mitigate strain rate effects induced by the loading rate, a rigid body was modeled atop the steel plate, with displacement control applied along the global vertical axis (Z-direction, U3 in ABAQUS notation).

### Material properties

#### Material modeling of concrete

The Concrete Damage Plasticity (CDP) model available in ABAQUS was employed to represent the nonlinear behavior of concrete. This model is suitable for materials exhibiting distinct tensile and compressive responses, as it separately evaluates damage mechanisms in tension and compression along the stress–strain path. The parameters used in the CDP model for this study are summarized in Table 3.

**Table 3:** Plastic damage parameters for CDP in ABAQUS.

Dilation angle	Eccentricity	F_b0_/f_c0_	K	Viscosity parameter
30.0	0.10	1.160	0.66667	0.0001

**Fig. 12:**
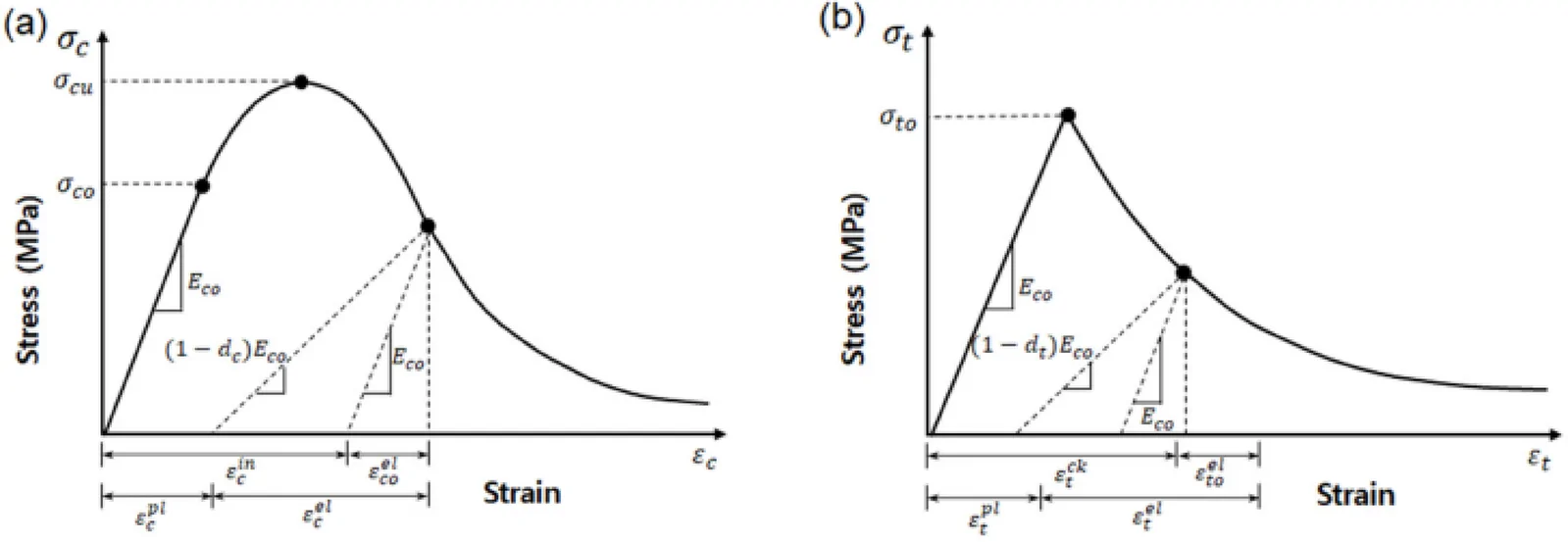
Concrete damage parameters: (**a**) compression, (**b**) tension

In addition, Fig. 12 displays the CDP parameters for concrete compression and tension in ABAQUS. The stress–strain diagram under non-elastic conditions describes the underlying concept governing the damage parameters.

The compression failure conditions ($\varepsilon_{c}^{in}$) and amount of concrete strain during cracking ($\varepsilon_{t}^{ck}$) were calculated using Eqs. (1)-(4) based on Fig. 12.

1 $$ \varepsilon_{c}^{in} = \varepsilon_{c} - \varepsilon_{c0}^{el} = \varepsilon_{c} - \frac{{\sigma_{c} }}{{\sigma_{c0} }} $$

2 $$ \varepsilon_{c}^{el} = \varepsilon_{c} - \varepsilon_{c}^{pl} = \varepsilon_{c} - b_{c} \varepsilon_{c}^{in} $$

3 $$ \varepsilon_{c}^{ck} = \varepsilon_{t} - \varepsilon_{t}^{ck} = \varepsilon_{t} - \frac{{\sigma_{t} }}{{E_{c0} }} $$

4 $$ \varepsilon_{t}^{el} = \varepsilon_{t} - \varepsilon_{t}^{pl} = \varepsilon_{t} - b_{t} \varepsilon_{t}^{in} $$

#### Reinforcement bars

**Fig. 13:**
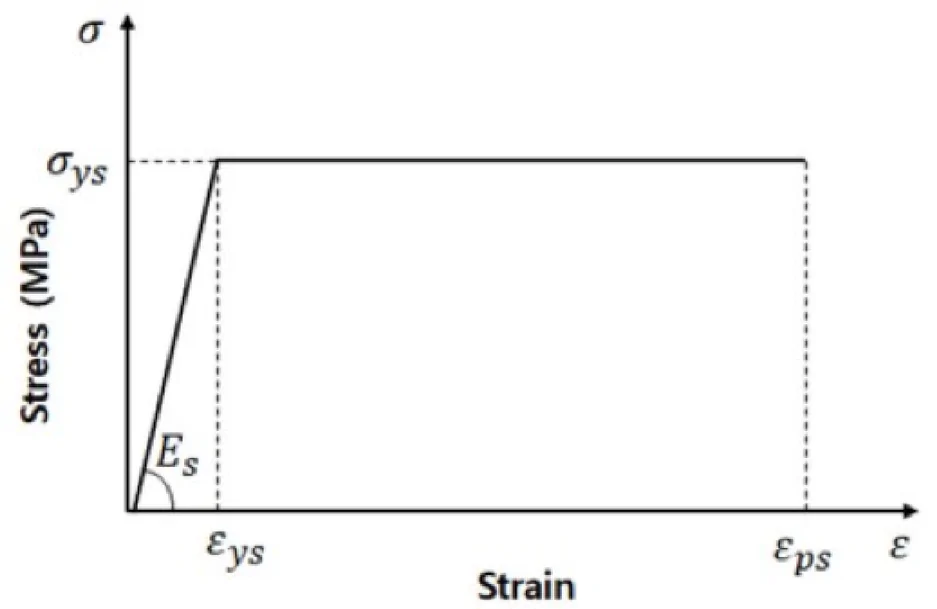
Stress–strain relationship for steel reinforcement bars

The reinforcing steel was modeled using a bilinear elastic–plastic stress–strain relationship, as illustrated in Fig. 13. The steel type utilized was Grade 36/52, characterized by an elastic modulus of 200 GPa, a yield strength of 361.2 MPa, and an ultimate strength of 530.1 MPa. A Poisson’s ratio of 0.3 was assigned for all reinforcement elements.

### Test results and analysis

**Fig. 14:**
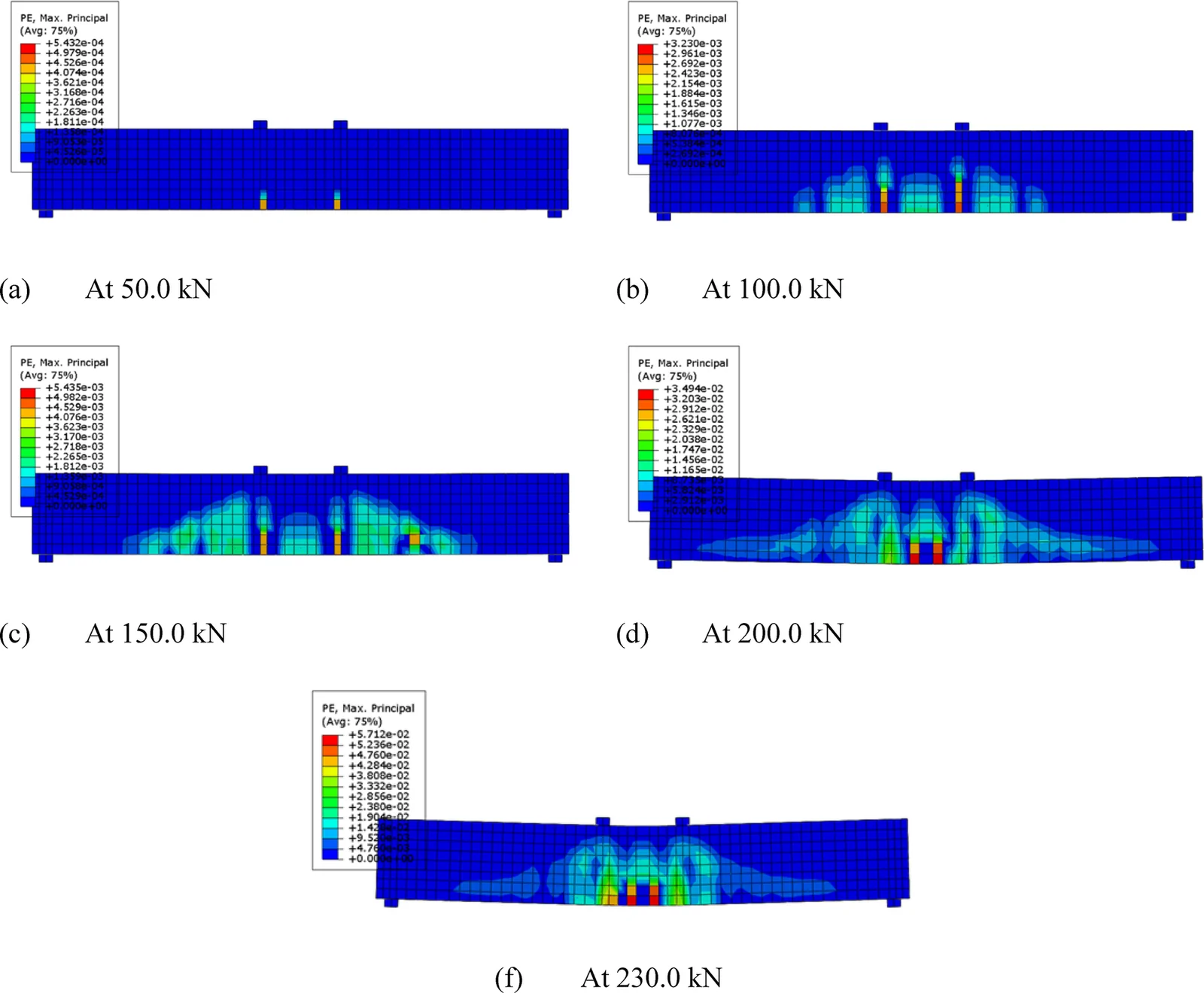
Plastic strain of RC beam at different load values

The ABAQUS simulation results indicated the occurrence of concrete crushing at the ultimate load level, as evidenced by significant nodal distortions in the mesh elements. This behavior is visually represented in Fig. 14, which illustrates the distribution of plastic strain under varying load conditions.

Table 4 presents the measured and corrected coordinates of the scanning points. The term Corrected Coordinates refers to the adjusted values obtained after alignment and error compensation in the laser scanning process. The vertical displacement (∆Z) increased nearly linearly with load up to around 150 kN. However, a deviation from linearity became evident at higher loads, particularly at 200 kN and more significantly at 230 kN, indicating a nonlinear structural response. Among all monitored points, Point 3 exhibited the greatest vertical deflection (approximately –26.1 mm at 230 kN), suggesting the onset of inelastic behavior and possibly marking areas of stress concentration or flexural softening.

**Table 4:** Measured and corrected coordinates of scanning points (after alignment procedure). Differences in coordinates represent deformations under applied load levels.

*P* = 0.0 kN			
Point ID	Coordinates		
	X (m)	Y (m)	Z (m)
1	0.258	0.3	0.165
2	0.849	0.3	0.173
3	1	0.3	0.168
4	1.127	0.3	0.161
5	1.713	0.3	0.160

Point ID	Differences in coordinates (deformation)		
	∆X(mm)	∆Y(mm)	∆Z(mm)
*P* = 50.0 kN			
1	− 0.000131	− 0.0015	− 0.00141
2	− 0.000131	− 0.00052	− 0.00163
3	− 0.000132	− 0.00132	− 0.00163
4	− 0.000132	− 0.00127	− 0.00162
5	− 0.000132	− 0.00169	− 0.00144
*P* = 100.0 kN			
1	− 0.00054	− 0.00054	− 0.00493
2	− 0.00053	− 0.00046	− 0.00404
3	− 0.00053	− 0.00054	− 0.0040
4	− 0.00052	− 0.00056	− 0.00403
5	− 0.00052	− 0.00058	− 0.00487
*P* = 150.0 kN			
1	− 0.00549	− 0.00512	− 0.00606
2	− 0.00535	− 0.00532	− 0.00791
3	− 0.00532	− 0.00547	− 0.00799
4	− 0.00528	− 0.00591	− 0.00796
5	− 0.00514	− 0.00595	− 0.00617
*P* = 200 kN			
1	− 0.000350	− 0.00079	− 0.00272
2	0.000617	− 0.00388	− 0.01357
3	0.00126	0.00011	− 0.0140
4	0.001960	− 0.00136	− 0.0136
5	0.00283	− 0.00106	− 0.00325
*P* = 230 kN			
1	− 0.0123	− 0.0128	− 0.0147
2	− 0.0114	− 0.0159	− 0.0256
3	− 0.0107	− 0.0119	− 0.0261
4	− 0.0100	− 0.0133	− 0.0256
5	− 0.0092	− 0.0130	− 0.0152

Furthermore, lateral displacements (∆X and ∆Y) were minimal under lower loads but became more noticeable at higher load levels, especially at 200 kN and above. This may reflect the emergence of lateral instability or the influence of geometric nonlinearities, such as second-order effects.

These findings highlight the value of incorporating nonlinear analysis in finite element simulations to better understand structural behavior near failure conditions. The ABAQUS model successfully captured both the onset and evolution of nonlinearity, offering valuable insight into deformation mechanisms under substantial loading.

## Numerical analysis using ANSYS

The numerical simulations were carried out using ANSYS Workbench 2023 R1 (ANSYS Inc., Canonsburg, PA, USA). The structural analysis module (Static Structural) was employed to simulate the four-point bending tests under static loading conditions. The ANSYS model was developed to reproduce the experimental four-point bending test using the same beam geometry, reinforcement arrangement, material properties obtained from the laboratory tests, support conditions, loading configuration, and loading increments. The analysis incorporated both material and geometric nonlinearities. The solution was performed incrementally using the Newton–Raphson iterative technique, and the numerical results were recorded after convergence was achieved. The predicted mid-span deflection was subsequently compared with the experimental measurements obtained from the dial gauge and TLS at identical load levels.

The following section discusses the specific modeling assumptions and results obtained from the FEM simulations. ANSYS, as a general-purpose FEM software, enables the simulation of complex scenarios, including linear and nonlinear static structural analysis, dynamic responses, thermal behavior, and fluid flow. In this study, deflection behavior of high-strength reinforced concrete beams was investigated using an FEM model implemented in ANSYS. The model incorporated crack development and deflection response under loading. The simulation was carried out incrementally using the Newton–Raphson iterative technique, with final results reported after achieving convergence.

The finite element models were developed to replicate the experimental program as closely as possible. The beam geometry, reinforcement layout, material properties obtained from laboratory tests, support conditions, loading arrangement, and loading increments were directly adopted from the experimental setup. Simply supported boundary conditions and the identical four-point loading configuration were implemented to ensure consistency between the experimental and numerical investigations.

### Finite element type and mesh

**Fig. 15:**
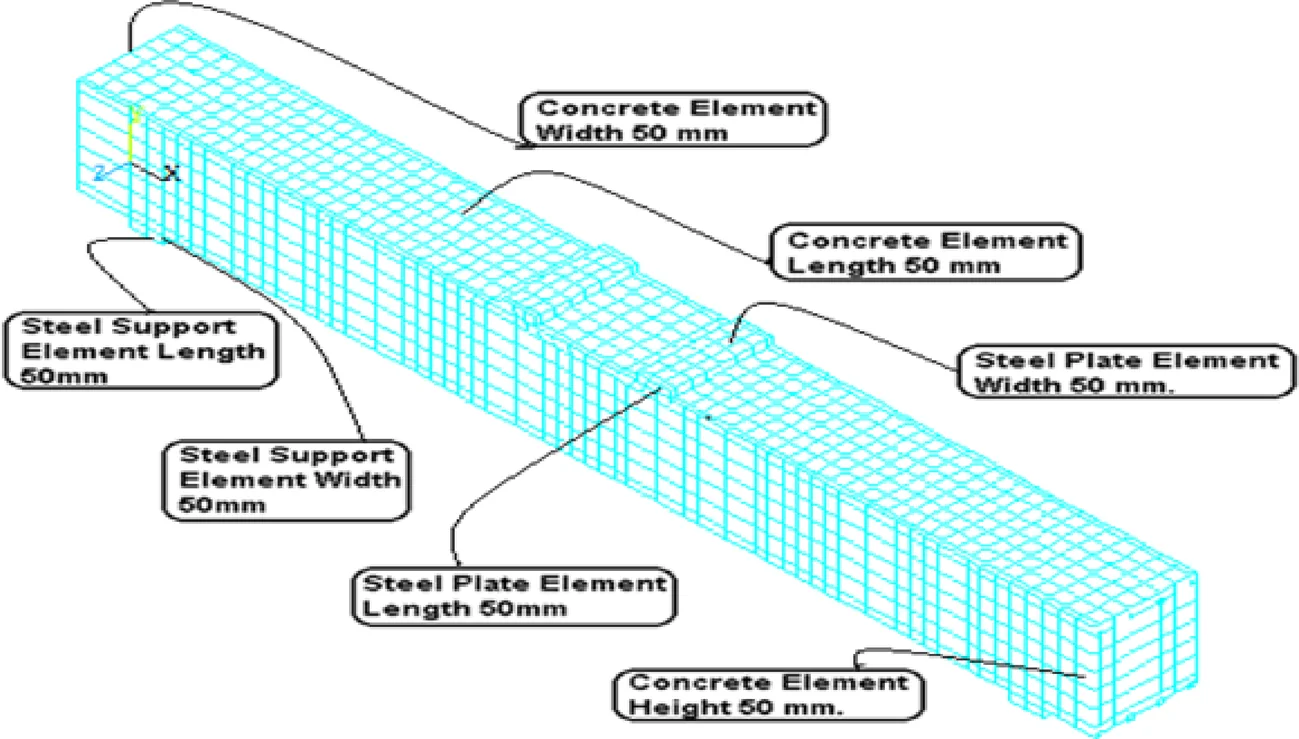
Element Types of ANSYS Modelling for the studied beam

**Fig. 16:**
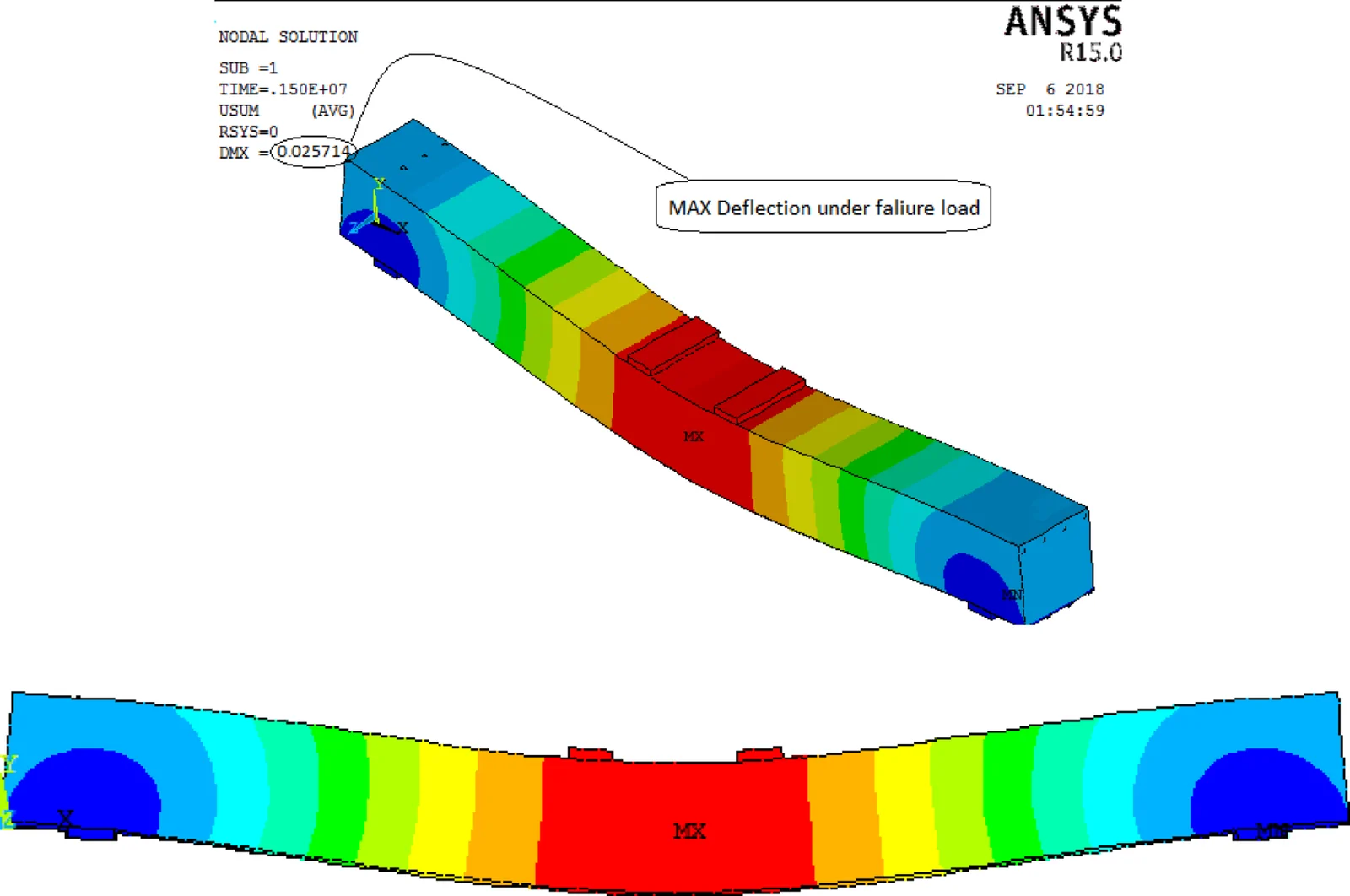
Max. deflection from ANSYS sheet for the studied beam

Selecting appropriate element types and mesh configurations is essential to ensure accurate representation of the beam’s behavior. A mesh refinement strategy was applied to balance accuracy and computational efficiency. The solid elements were assigned aspect ratios ranging between 1 and 3, adhering to acceptable modeling standards. The simulation incorporated both material and geometric nonlinearities. The concrete was represented using the SOLID65 element, an 8-node element specifically designed to capture the nonlinear behavior of brittle materials, such as cracking and crushing. Each node possesses three translational degrees of freedom. Prior to damage, concrete was considered isotropic and elastic. Upon cracking, its behavior transitioned to orthotropic, with directional stiffnessgoverned by a bilinear softening model in the crack normal direction. Steel reinforcement was represented using the LINK180 element, which is a uniaxial, 2-node bar element capable of simulating plastic deformation. These elements were embedded in the concrete and shared common nodes to ensure proper interaction. Additionally, steel loading plates were modeled using SOLID185 elements to mitigate premature failure due to localized stress concentrations at the supports and loading zones. Figure 15 illustrates the three-dimensional FEM mesh of the beam, including reinforcement and boundary supports. The beam geometry used in the finite element model was identical to that described in “Experimental work using TLS” section. Zero values for the Z-coordinate represent the beam’s mid-depth The use of the Static Structural analysis system together with the nonlinear SOLID65 concrete formulation, LINK180 reinforcement elements, and SOLID185 loading plates allowed the numerical model to reproduce the principal nonlinear response mechanisms observed experimentally, including concrete cracking, reinforcement deformation, and the resulting beam deflection under increasing load.

, as shown in Fig. 16.

### Material modeling

**Fig. 17:**
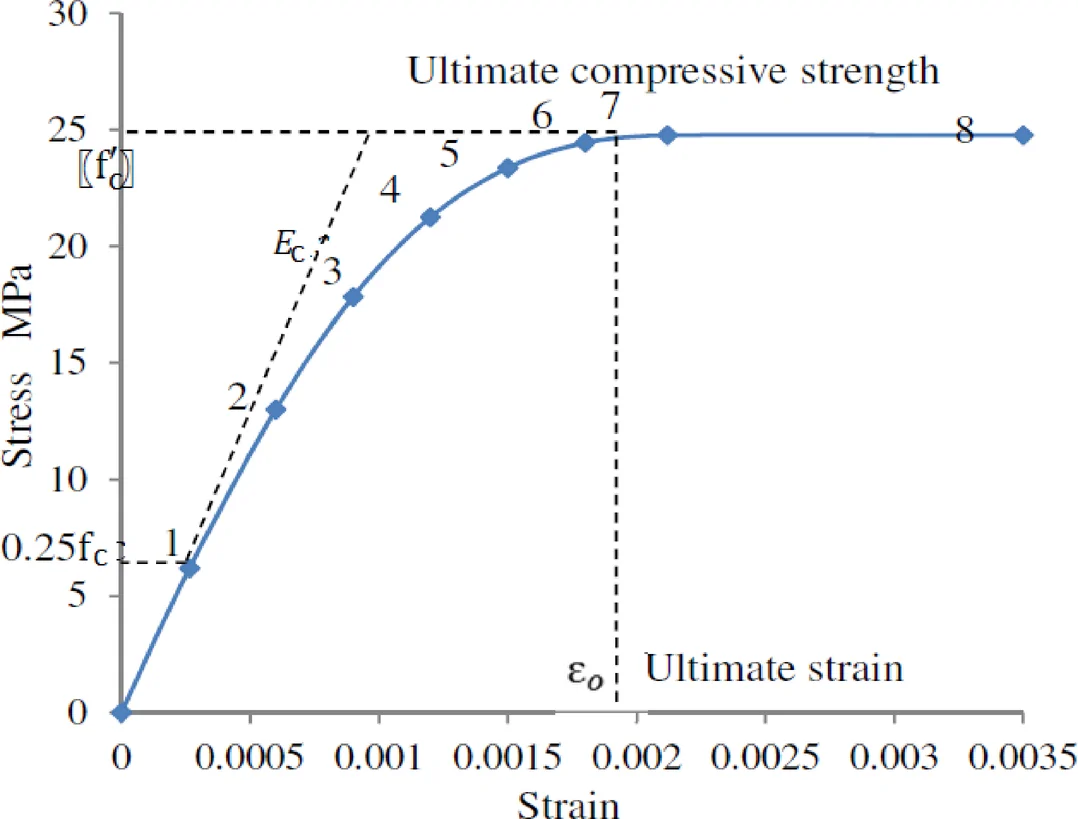
Stress- Strain curve for concrete

Material properties were defined based on the experimentally determined properties of the concrete and reinforcement. The ultimate concrete strain was set to 0.003, with a Poisson’s ratio of 0.2. Concrete under compression was modeled using a multi-linear isotropic stress–strain relationship. The equations used to define the concrete stress–strain relationship were adopted from the established formulations reported in the relevant literature and were not newly developed by the authors. Equations (1)–(3) were used to define the basic stress–strain relationship and the strain corresponding to the peak stress, while Eqs. (5)–(7) were used to define the ascending branch of the concrete stress–strain curve. The adopted equations were used consistently with the material modeling procedure implemented in the finite element analysis. To ensure numerical convergence, the descending branch of the concrete stress–strain curve was omitted in accordance with the adopted modeling approach. Steel reinforcement behavior was represented using a bilinear stress–strain relationship, with a Poisson’s ratio of 0.3. Loading and support plates were assigned linear elastic material properties. Figure 17 presents the resulting stress–strain curve used for the concrete material model.

5 $$ f = \frac{{E_{c} \varepsilon }}{{1 + \left( {\frac{\varepsilon }{{\varepsilon_{o} }}} \right)}} $$

6 $$ \varepsilon_{o} = \frac{{2f_{c}^{{\prime }} }}{{E_{C} }} $$

7 $$ E_{c} = \frac{f}{\varepsilon } $$

### Boundary condition and load application

At the point of edge support, simple supported boundary conditions were used. Only half of each beam was modelled due to the symmetry of all the pre-tested beams, as shown in Fig. 15. The boundary conditions were defined to replicate the experimental setup. One end of the beam was modeled as a hinged support, restraining displacements in both the X and Z directions while allowing rotation. The opposite end was modeled as a roller support, restraining vertical displacement (Z-direction) but permitting free movement in the horizontal (X) direction to prevent axial restraint. Additionally, nodes along the mid-plane of symmetry were restrained in the out-of-plane direction to ensure numerical stability. These constraints ensured realistic load transfer while avoiding artificial stiffening of the model. The cracking and crushing of concrete elements are monitored until the failure load.

### Steel plates

To minimize stress concentrations and prevent localized crushing of the concrete near the support and load application areas, a 15 mm-thick steel plate was placed at the support locations. These plates were modeled using SOLID185 elements to ensure a uniform stress distribution across the support region. Each SOLID185 element comprises eight nodes, with each node having three translational degrees of freedom in the x, y, and z directions, as defined in ANSYS.

### Test results and analysis

Table 5 shows the displacement results obtained from the ANSYS simulation for five selected points under incremental loads up to 230 kN illustrate the structural deformation behavior in detail.

**Table 5:** The adjusted coordinates and deformation of the monitoring points.

*P* = 0.0 kN			
Point ID	Coordinates		
	X (m)	Y (m)	Z (m)
1	0.24865	0.3	0.174681
2	0.74938	0.3	0.18312
3	0.98542	0.3	0.17841
4	1.11062	0.3	0.17154
5	1.21519	0.3	0.17026

Point ID	Differences in coordinates (deformation)		
	∆X	∆Y	∆Z
*P* = 50.0 kN			
1	7.67387E−06	− 0.00019456	− 0.00010354
2	3.30953E−06	0.00079139	− 0.00031891
3	1.74262E−08	− 5.0896E−06	− 0.00089527
4	− 1.5276E−06	4.84102E−05	− 0.0003084
5	− 5.15929E−06	− 0.00037518	− 0.00012237
*P* = 100.0 kN			
1	− 5.58505E−05	− 2.50549E−05	− 0.000380612
2	− 1.79455E−05	0.000739797	− 0.00127286
3	− 3.45447E−07	− 7.19461E−05	− 0.00211105
4	2.77909E−05	− 0.000275013	− 0.00128353
5	0.000064832	− 0.000468772	− 0.000441161
*P* = 150.0 kN			
1	− 0.000177246	0.000200842	− 0.000741547
2	− 3.21778E−05	− 6.74983E−06	− 0.00358912
3	− 6.58054E−06	− 0.000153282	− 0.00467872
4	3.71115E-05	− 0.000593635	− 0.00364401
5	0.000176648	− 0.000638429	− 0.000853849
*P* = 200 kN			
1	− 0.001166	− 0.00160171	− 0.00353545
2	− 0.000198454	− 0.00470469	− 0.0113922
3	0.000440637	− 0.000705156	− 0.0128153
4	0.00114527	− 0.00217306	− 0.0114324
5	0.00202018	− 0.00187906	− 0.0040681
*P* = 230 kN			
1	− 0.005162	− 0.00760181	− 0.00353545
2	− 0.000297482	− 0.00870445	− 0.0143922
3	0.000740434	− 0.000902186	− 0.0218153
4	0.00514329	− 0.00418302	− 0.0164324
5	0.00502419	− 0.00687808	− 0.0016068

#### Initial configuration (P = 0 kN):

The coordinates represent the initial unreformed geometry of the structure, serving as a baseline for displacement calculations.

#### Low to moderate loads (P = 50 kN to 150 kN):

Displacements remain relatively small and mostly linear in nature. Vertical displacements (∆Z) gradually increase with load, reaching up to approximately − 4.68 mm at 150 kN (Point 3), indicating elastic bending behavior. Lateral displacements (∆X and ∆Y) are minimal, staying within the sub-millimeter range and showing no significant trends at these load levels.

#### Higher loads (P = 200 kN):

At 200 kN, vertical displacements significantly increase, with Point 3 experiencing about − 12.82 mm displacement in the Z-direction. Lateral displacements also become more pronounced, especially in the Y-direction, where displacements reach up to approximately − 4.7 mm at Point 2. This lateral shift indicates the onset of second-order effects and geometric nonlinearities, as the beam no longer deforms solely in the vertical (Z) direction. Such behavior can be attributed to the combined influence of shear deformations around the web openings and the reduction of stiffness under increasing load, which promotes out-of-plane displacement. This observation highlights the transition from a primarily flexural response to a coupled flexural–lateral response, which is critical in assessing stability prior to failure..

#### Near ultimate load (P = 230 kN):

The structure demonstrates a further increase in displacement magnitudes. The highest vertical displacement is recorded at Point 3 (≈ − 21.82 mm), indicating significant deflection that may correspond to inelastic behavior or localized damage. Lateral displacements increase notably, with Point 4 showing a positive displacement of approximately 5.14 mm in the X-direction and several points showing larger negative displacements in Y, suggesting lateral instability effects.

ANSYS simulation results display a predominantly linear-elastic displacement response up to 150 kN, beyond which nonlinear effects become evident. The increase in lateral displacements and the large vertical deflections near 230 kN highlight the importance of considering geometric and material nonlinearities in structural analyses approaching ultimate load states. These numerical findings provide crucial insight into the progressive deformation and potential failure mechanisms of the structure.

## Comparison between TLS observations and other techniques

To validate the proposed methodology, TLS measurements were quantitatively compared with dial gauge readings, total station observations, analytical calculations, and finite element predictions. Validation was assessed based on the absolute differences in measured deflections under identical loading stages. The close agreement among these independent techniques was considered evidence of the reliability and repeatability of the TLS measurements.

Table 6 presents a comparison of vertical deflections (∆Z) measured experimentally using Terrestrial Laser Scanning , Total Station, and Dial Gauge, alongside theoretical predictions and numerical results obtained from ANSYS and ABAQUS simulations under varying load levels.

**Table 6:** Maximum deflection of the tested RC beam (mm).

Load (kN)	∆ (TLS)	∆ Total station	∆ Theoritical	∆ ANSYS	∆ ABAQUS	∆ Dial gauge
0.0	0.0	0.0	0.0	0.0	0.0	0.0
50	2.0	1.8	1.8	0.9	1.6	2.1
100	3.0	2.7	2.7	2.1	4.0	3.2
150	7.0	7.2	6.5	4.7	8.0	7.1
200	14.0	14.1	13.2	12.8	14.1	14.2
230	26.0	25.8	25.7	21.8	26.1	26.3

A statistical comparison was performed using the deflection measurements obtained at the common loading levels. The differences between TLS and each reference method were evaluated using the mean absolute error (MAE), root mean square error (RMSE), and mean absolute percentage error (MAPE). These metrics were selected to quantify both the average magnitude of the deviations and the relative differences between the TLS measurements and the corresponding reference results. The statistical comparison was performed using the available common load levels and was therefore interpreted as an assessment of agreement within the experimental loading range rather than as a population-level statistical inference.

To provide a quantitative assessment of the agreement among the different approaches, the TLS measurements were compared with the dial gauge, total station, theoretical calculations, ANSYS, and ABAQUS results at identical load levels. The comparison was assessed using the absolute difference and relative percentage difference between each method and the TLS measurements. This quantitative assessment provides an objective basis for evaluating the consistency of the independent experimental measurements and the agreement of the analytical and numerical predictions with the experimentally observed deformation response. Particular attention was given to the higher load levels, where cracking and nonlinear material behavior become more pronounced and differences between the numerical predictions and experimental measurements may increase.From Table 6, The data reveal good agreement between the different measurement techniques and numerical models, especially at lower to moderate loads. At 50 kN, displacements measured by TLS (0.0020 m) and Dial Gauge (0.0021 m) closely align, while numerical predictions from ABAQUS .

The difference between TLS and Dial Gauge at ultimate load was only 0.3 mm, demonstrating excellent agreement. (0.00163 m) and ANSYS (0.00090 m) slightly underestimate the deflection. As the load increases, the measured displacements from TLS and Total Station show a consistent and nearly linear increase up to 230 kN, reaching approximately 0.026 m, which is closely mirrored by the Dial Gauge readings (0.0263 m). The theoretical displacement values also follow this trend but tend to slightly underestimate the response at higher loads, likely due to idealizations in the theoretical model. Table 7 shows the variance between maximum deflection from TLS observation and other different techniques.

**Table 7:** Differences between all applied techniques and TLS.

Load (kN)	∆ (TLS)	*l*_*2*_*-l*_*1*_	*l*_*3*_*-l*_*1*_	*l*_*4*_*-l*_*1*_	*L*_*5*_*-l*_*1*_	*L*_*6*_*-l*_*1*_
0.0	0.0	0.0	0.0	0	0	0.0
5.0	0.0020	− 0.00020	− 0.000160	− 0.0001047	0.00063054	0.0001
10.0	0.0030	− 0.00030	− 0.000320	− 0.0018889	2.31E−06	0.0002
15.0	0.0070	0.00020	− 0.000470	− 0.0033213	-2.1E−06	0.0001
20.0	0.0140	0.00010	− 0.000770	− 0.001185	5.41E−05	0.0002
23.0	0.0260	− 0.00020	− 0.000270	− 0.004185	0.0001052	0.0003

**Fig. 18:**
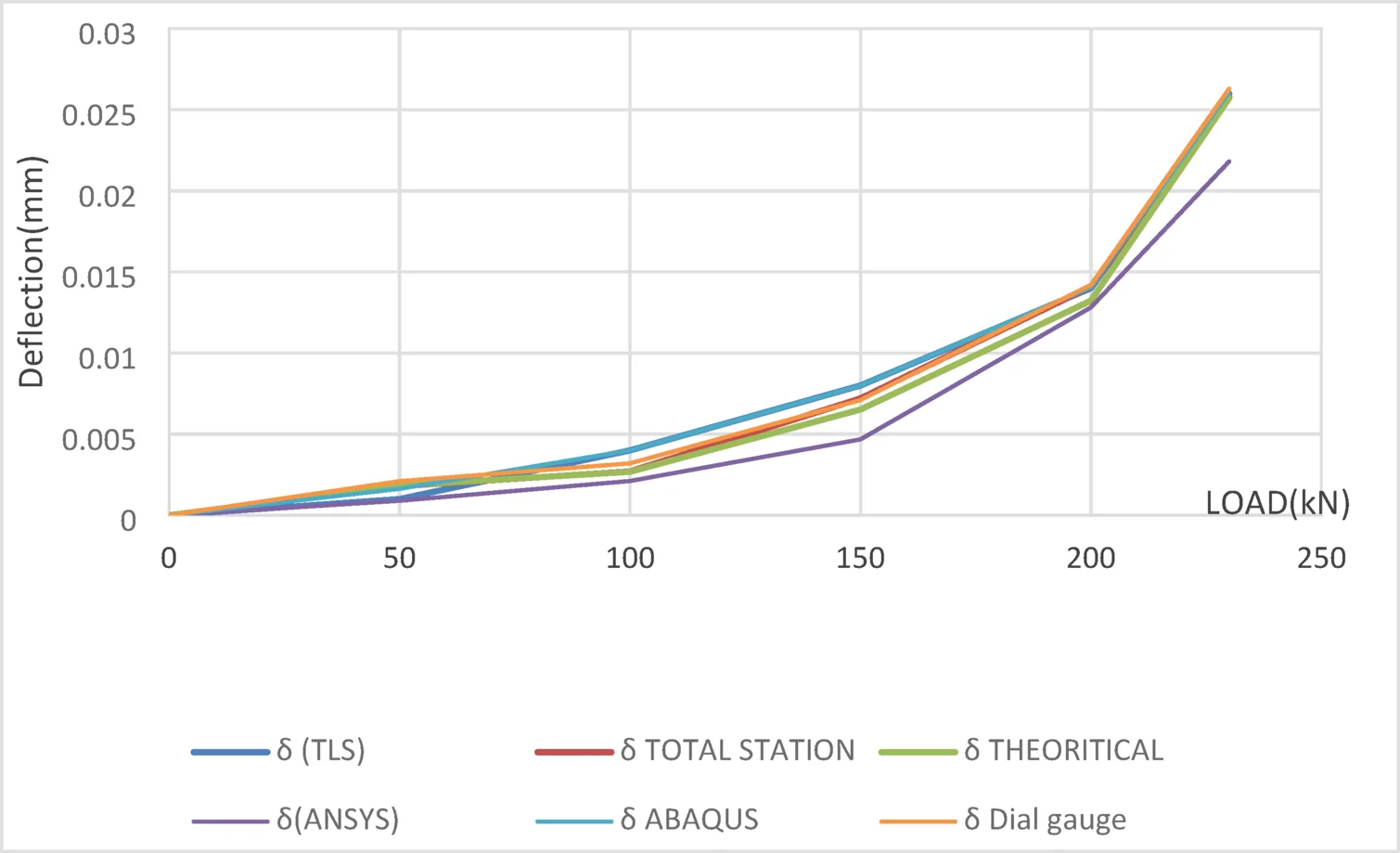
Max. deflection for all techniques applied

As shown in Table 7 and Fig. 18, Numerical results from ANSYS and ABAQUS capture the overall deformation behavior well; however, ABAQUS predictions tend to be closer to the experimental measurements than ANSYS at higher load levels, with ANSYS slightly underestimating deflections near ultimate load (230 kN). This discrepancy may arise from differences in modeling assumptions, material nonlinearity, or boundary conditions.

Overall, the comparison validates the accuracy of the numerical models and experimental techniques, emphasizing the importance of integrating multiple approaches for reliable structural deformation assessment, particularly near ultimate load capacity where nonlinear effects become significant.

## Conclusion

Monitoring deformation in reinforced concrete structures is a critical aspect for ensuring their safety and long-term performance. This study aims to evaluate the effectiveness of using Terrestrial Laser Scanning (TLS) as a modern, non-invasive tool for accurately determine the deformation of RC beams. Based on the findings of this research, the following conclusions can be drawn:

- This study has demonstrated the effectiveness of using Terrestrial Laser Scanning as a precise and reliable geodetic technique for monitoring the deformation in reinforced concrete beams under static loading. Experimental results show that TLS measurements are highly consistent with traditional tools such as dial gauges and total stations, as well as numerical simulations from ANSYS and ABAQUS.
- Among the studied techniques and methods, ABAQUS and TLS provided the closest results to the physical measurements using dial gauges, indicating their superior capability for deformation tracking in structural health monitoring applications.
- TLS is a quick acquisition method that does not need deploying targets on the object. Because the measurements are performed without touching the object, their performance and accuracy are determined by its surface qualities.
- The analysis of mid-span deflection values measured by different methods under increasing loads up to 230 kN shows that at maximum load, the dial gauge recorded 26.3 mm deflection. TLS and ABAQUS gave a very close reading of 26 mm, while ANSYS simulations predicted about 25.72 mm. The Total Station and theoretical calculations showed 25.8 mm and 25.72 mm, respectively. These consistent results, with differences within ± 0.3 mm, confirm that TLS and ABAQUS is accurate and reliable for monitoring structural deformation, performing on par with traditional instruments and numerical models.
- TLS is regarded as a good method for precisely monitoring the deformation of structural parts. It can also construct 3D representations of monitored objects through loading.

## Data Availability

The datasets generated and/or analyzed during the current study are available from the corresponding author on reasonable request.
